# Supplementary material for: Changes in Fat Mass Following Creatine Supplementation and Resistance Training in Adults ≥50 Years of Age: A Meta-Analysis
Source: J Funct Morphol Kinesiol. 2019 Aug 23;4(3):62. doi: 10.3390/jfmk4030062 (PMC7739317; doi:10.3390/jfmk4030062)
Supplement: Supplementary file 1 [file jfmk-04-00062-s001.pdf]

Table S1: Individual study characteristics.

First Author, Year	Population	Supplement Dose	Resistance Training and Duration	Training Volume	Dietary Intake	Body Composition Tool	Outcomes
Anguiar et al., 2013	N=18; healthy women; Mean age=65 years	CR (5g/day) or PLA	RT = 3 days/wk; 12 weeks	CR ↑ training volume compared to placebo	↔ % CHO, % Protein, %Fat before or after the training.	DEXA	CR ↑ gains in fat-free mass (+3.2%) ↔ on body mass, fat mass, and % body fat over time or between groups.
Bermon et al., 1998	N=32 (16 men, 16 women); healthy; age=67-80 years	CR (20g/day for 5 days followed by 3 g/day) or PLA	T = 3 days/wk; ~7.4 weeks (52 days)	Not Reported	Only reported as normal (mini nutritional assessment).	Skinfold Thickness.	↔ body mass, body fat over time or between groups.
Brose et al., 2003	N=28 (15 men, 13 women); healthy; age: men = 68.7, women = 70.8 years	CR (5g/day) or PLA	RT = 3 days/wk; 14 weeks	Not Reported	↔ for energy intake or macronutrient over time or between groups.	DEXA	CR ↑ gains in lean tissue mass and total body mass ↔ in % body fat or fat mass over time or between groups.
Candow et al., 2008	N=35; healthy men; age=59-77 years	CR (0.1g/kg/day) or CR+protein (0.3g/kg/day) or PLA	RT = 3 days/wk; 10 weeks	↔ on training volume between groups.	↔ for energy intake or macronutrient over time or between groups.	Air-displacement plethysmography	CR and CR+protein conditions combined ↑muscle thickness compared to PLA. CR ↑body mass compared to PLA. ↔ % body fat or fat mass.
Candow et al., 2015	N=39 (17 men, 22 women); healthy; age = 50-71 years	CR (0.1g/kg) before RT, CR (0.1 g/kg) after RT, or PLA	RT = 3 days/wk; 32 weeks	↔ on training volume between groups.	↑ energy intake over time; ↔ between groups.	DEXA	Cr After ↑ lean tissue mass compared to PLA. ↔ Fat mass between groups (sig. decrease over time: main effect).
Chilibeck et al., 2015	N=33; healthy women; Mean age=57 years	CR (0.1g/kg/day) or PLA	RT=3 days/wk; 52 weeks	↔ on training volume between groups. Sig. time effect.	↔ Energy between groups or over time.	DEXA	↔ lean tissue mass, body mass, fat mass, or percent body fat.

Chrusch et al., 2001	N=30; healthy men; age=60-84 years	CR (0.3g/kg/d for 5 days followed by 0.07 g/kg/day) or PLA	RT = 3 days/wk; 12 weeks	CR ↑ training volume (31% greater) than placebo.	Not Reported	DEXA	CR ↑ gains in lean tissue mass and body mass ↔ % body fat or fat mass over time or between groups.
Collins et al., 2016	N=16 (frail men and women); age=70 years	CR (4 g/day) and protein (20g/day) or protein	RT = 2 days/wk; 14 weeks	Not Reported	↔ energy or protein between groups (time effect)	DEXA	↔ body mass, fat-free mass, or fat mass.
Cooke et al., 2014	N=20; healthy men; age=55-70 years	CR (20g/day for 7 days than 0.1 g/kg/day on training days)	RT = 3 days/wk; 12 weeks	↔ in training volume between groups. ↑ main effect over time.	↔ energy or protein between groups (time effect)	DEXA	↔ body mass, lean tissue mass. CR ↓ (trend) % body fat and absolute fat mass.
Deacon et al., 2008	N=80 (50 men, 30 women); COPD; age=68.2 years	CR (22g/day for 5 day followed by 3.76g/day) or PLA	RT = 3 days/wk; 7 weeks	Not Reported	Not Reported	Bioelectrical impedance	↔ lean tissue mass or body mass between groups (both sig. increased over time). ↔ fat mass over time or between groups.
Eijnde et al., 2003	N=46; healthy men; age=55-75 years	CR (5g/day) or PLA	Cardiorespiratory + RT = 2-3 days/wk; 26 weeks	↔ in training volume between groups.	Not Reported	Hydrostatic Weighing	↔ body mass, % body fat, and fat-free mass compared to PLA.
Eliot et al., 2008	N=42; healthy men; age=48-72 years	CR (5g/day), protein (35 g/day), CR+protein, or PLA	RT = 3 days/wk; 14 weeks	Not Reported	↔ Energy between groups.	DEXA	↔ % body fat, total body fat, or body mass compared to PLA.
Gualano et al., 2011	N=25 (9 men, 16 women); type 2 diabetes; age=57 years	CR (5 g/day) or PLA	RT = 3 days/wk; 12 weeks	↔ in training volume between groups.	↔ Energy between groups.	DEXA	↔ body mass, fat mass, or lean tissue mass

Gualano et al., 2014	N=30; "vulnerable" women; Mean age=65.4 years	CR (20g/day for 5 days; 5 g/day thereafter) or PLA with and without RT	RT=2 days/wk; 24 weeks	Not Reported	↔ Energy and macronutrient intake between groups.	DEXA	CR ↑ appendicular lean mass compared to PLA. ↔ Body mass or fat mass over time or between groups.
Johannsmeyer et al., 2016	N=31 (17 men, 14 women); healthy; age=58 years	CR (0.1g/kg/day) or PLA	RT = 3 days/wk; 12 weeks	↔ in training volume between groups.	↔ Energy and macronutrient intake between groups.	DEXA	CR ↑ body mass and lean tissue mass compared to PLA. ↓ body fat over time ↔ between groups.
Neves et al., 2011	N=24 (postmenopausal women with Knee osteoarthritis); Age=55-65 years	CR (20 g/day for 1 week, followed by 5 g/day) or PLA	RT=3 days/wk; 12 weeks	Not Reported	↔ Energy between groups or over time.	DEXA	CR ↑ limb lean mass. ↔ body mass or % body fat. % body fat ↓over time.
Pinto et al., 2016	N=27 (men and women); healthy; age=60-80 years	CR (5 g/day) or PLA	RT = 3 days/wk; 12 weeks	Not Reported	↔ Energy between groups or over time.	DEXA	CR ↑ gains in body mass and lean tissue mass; CR ↓body fat compared to PLA ($p=0.053$).
Tarnopolsky et al, 2007	N=39 (19 men, 20 women); healthy; age=65-85 years	CR (5 g/day) + CLA (6g/day) or PLA	RT = 2 days/wk; 26 weeks	Not Reported	↔ Energy between groups or over time. Men had greater energy intake than women.	DEXA	CR + CLA ↑ gains in lean tissue mass; CR + CLA↓ fat mass compared to PLA; ↔ body mass or fat mass.
Villaneueva et al., 2014	N=14; healthy men; age=68.7 years	CR (0.3 g/kg/day for 5 days followed by 0.07 g/kg/day) + 35 g protein or PLA	RT = 3 days/wk; 12 weeks	Not Reported	↔ Energy between groups.	DEXA	↔ lean tissue mass, fat mass, or % body fat. CR + Protein ↓ % body fat compared to baseline.

RT = resistance training; CR = Creatine; PLA = Placebo; CLA = conjugated linoleic acid; DEXA = dual energy x-ray absorptiometry
